# Antioxidant Effects of *Thymus vulgaris* Seed Oil on Paracetamol-Induced Oxidative Alterations in Mice Liver Tissue

**DOI:** 10.3390/plants15213404

**Published:** 2026-11-02

**Authors:** Jelena Živković, Dejan Pljevljakušić, Hanaa Salah, Katarina Šavikin, Dragana Vujanović, Tatjana Ćebović

**Affiliations:** 1 Institute for Medicinal Plants Research “Dr Josif Pnčić”, Tadeuša Košćuška 1, 11000 Belgrade, Serbia; dpljevljakusic@mocbilja.rs (D.P.); ksavikin@mocbilja.rs (K.Š.); 2 Department of Medical Laboratory Techniques, Al-Farahidi University, Baghdad 10021, Iraq; hanaa.salim@uoalfarahidi.edu.iq; 3 Department of Pharmacy, Singidunum University, Danijelova 32, 11000 Belgrade, Serbia; dvujanovic@singidunum.ac.rs; 4 Department of Biochemistry, School of Medicine, University of Novi Sad, 21102 Novi Sad, Serbia; tatjana.cebovic@mf.uns.ac.rs

**Keywords:** *Thymus vulgaris*, seed oil, α-linolenic acid, carotenoids, tocopherols, hepatoprotection, oxidative stress

## Abstract

*Thymus vulgaris* seed oil represents an unexplored source of bioactive lipophilic compounds with potential health-promoting properties. This study aimed to characterize the fatty acid, carotenoid, and tocol composition of *T. vulgaris* seed oil and investigate its effects on hepatic oxidative stress parameters in a mice model of paracetamol-induced toxicity. Fatty acid analysis revealed a predominance of polyunsaturated fatty acids (79.0%), with α-linolenic acid as the major constituent (63.3%), followed by 2-hydroxy-linolenic acid (10.5%) and linoleic acid (4.9%). Among carotenoids, lutein was identified as the predominant pigment, followed by β-carotene. The oil was also characterized by a high content of vitamin E compounds, with γ-tocopherol and γ-tocotrienol representing the dominant tocols. The in vivo study included 48 NMRI Hannover mice randomly assigned to eight experimental groups (*n* = 6 per group; three males and three females). The mice received *T. vulgaris* seed oil at doses of 1, 2, or 3 mL/kg body weight orally for seven days, with or without paracetamol administration (300 mg/kg, intraperitoneally) on day 7. Liver samples were collected 24 h after paracetamol administration to assess oxidative stress parameters, including superoxide dismutase (SOD), catalase (CAT), glutathione reductase (GR), glutathione peroxidase (GSH-Px), reduced glutathione (GSH), xanthine oxidase (XOD), and lipid peroxidation (LPx). Oral administration of *T. vulgaris* seed oil significantly improved hepatic antioxidant defence parameters in a dose-dependent manner. The highest tested dose (3 mL/kg b.wt.) produced the most pronounced effects, restoring antioxidant enzyme activities, increasing GSH content, and reducing XOD activity and lipid peroxidation toward the values observed in non-intoxicated animals. The observed biological activity may be associated with the presence of ω-3 polyunsaturated fatty acids, carotenoids, and vitamin E compounds, which may contribute to antioxidant defence and the protection of cellular membranes against oxidative damage. These findings indicate that *T. vulgaris* seed oil may mitigate hepatic oxidative stress in an acute mouse model of paracetamol-induced toxicity, although further studies are needed to confirm these effects and clarify their underlying mechanisms.

## 1. Introduction

Liver diseases represent a global health challenge, since the liver is the main organ responsible for metabolism, detoxification, and xenobiotic biotransformation. There are numerous causes of liver damage (viruses, alcohol, drugs—Drug-Induced Liver Injury—DILI, industry and environmental chemicals, metabolic syndrome—non-alcoholic fatty liver disease—NAFLD). Many chemicals, as well as environmental toxic substances, can cause liver damage: chloroform, benzene, polychlorinated biphenyls (PCBs), heavy metals (arsenic, copper, iron in toxic amounts), poisonous mushrooms (e.g., *Amanita phalloides*, which contains alpha-amanitin), etc. [1]. Regardless of the primary cause, liver damage is mainly caused by the generation of reactive oxygen species (ROS) and the induction of lipid peroxidation, leading to hepatocyte inflammation, fibrosis, and necrosis [2,3]. According to the World Health Organization (WHO), an estimated 287 million people were living with chronic hepatitis B or C worldwide in 2024, and approximately 1.34 million deaths were attributed to these infections, primarily through cirrhosis and hepatocellular carcinoma [4]. In addition to these chronic conditions, drug-induced liver injury represents an important cause of acute hepatic damage. Recent reviews estimate its incidence at approximately 14–19 cases per 100,000 individuals, while paracetamol overdose remains a leading cause of acute liver failure in several Western countries [5].

In this study, we examined the antioxidant potential of oral administration of *T. vulgaris* seed oil in a rat model of paracetamol-induced liver injury. Although typically safe when used at recommended therapeutic doses, the excessive ingestion of paracetamol as an over-the-counter drug can potentially cause hepatotoxicity, which may lead to acute liver failure [6]. Interest in fatty oils obtained from the seeds of different nutritional and medicinal plants as a functional ingredient with antioxidant potential has grown in recent years [7]. Fatty oils are complex mixtures of fatty acids, glycerides and minor lipophilic constituents such as tocopherols (mainly vitamin E homologues), carotenoids and some phenolic esters. It was shown that unsaturated fatty acids can modulate membrane fluidity and cell signaling, while tocopherols could ameliorate lipid peroxidation and, together with carotenoids, could scavenge peroxyl radicals [8,9]. Due to this, they can stabilize membranes, preserve endogenous GSH pools, and support antioxidant enzyme activities. Seed oils and enriched lipid fractions have been evaluated as dietary ingredients capable of reducing oxidative liver injury and, in particular, modulating metabolic changes connected to non-alcoholic fatty liver disease and xenobiotic-induced toxicity [10].

Thyme (*Thymus vulgaris*, Lamiaceae) has a long ethnobotanical tradition and is used in nutrition and for medicinal purposes due to its expectorant, hepatoprotective, fungicidal, antiviral, anti-inflammatory, and other properties [11]. It is also widely studied for its essential oil rich in phenolic monoterpenes (e.g., thymol, carvacrol) with significant antimicrobial activity [12,13]. Moreover, recent phytochemical studies showed that both extracts and essential oils obtained from *Thymus* species could have beneficial activity in some conditions and diseases, including hepatoprotection [14]. While the chemistry and biological activities of essential oil and different extracts of *T. vulgaris* aerial parts have been extensively reported, much less attention has been paid to the chemical composition and biological properties of *T. vulgaris* seed oil, a non-volatile lipid fraction that contains a distinct fatty acid profile together with lipophilic antioxidants such as tocopherols and carotenoids. Al-Assaf et al. [15] reported DPPH radical scavenging activity of fatty oil extracted from seeds of *Thymus vulgaris* harvested in Iraq, which was comparable to quercetin (82.60% and 85.32%, respectively).

This study aimed to evaluate the antioxidant potential of *Thymus vulgaris* seed oil in a rat model of paracetamol-induced liver injury by assessing its ability to preserve endogenous glutathione (GSH) levels; normalize superoxide dismutase (SOD), catalase (CAT), and glutathione reductase (GR) activities; and modulate lipid peroxidation (LPx), glutathione peroxidase (GSH-Px), and xanthine oxidase (XOD) activity. In addition, the oil was chemically characterized by analyzing its fatty acid profile and determining its tocopherol and carotenoid contents.

## 2. Results and Discussion

### 2.1. Analysis of Fatty Acids

In the seed fatty oil of *Thymus vulgaris*, polyunsaturated fatty acids (PUFAs) dominated the profile, accounting for 79.0% of the total fatty acid fraction (Table 1). The most abundant component was α-linolenic acid, representing 63.3%, followed by 2-hydroxy-linolenic acid (10.5%) and linoleic acid (4.9%). Monounsaturated fatty acids (MUFAs) constituted the second most abundant group (13.4%), with oleic acid (10.6%) and heptadecenoic acid (2.3%) as the primary representatives. Saturated fatty acids (SFAs) were least represented (6.7%), almost entirely composed of palmitic acid (4.3%) and stearic acid (2.1%).

The predominance of PUFAs observed in our study is consistent with previously reported lipid profiles of several *Thymus* species, where α-linolenic acid (ALA) is commonly the dominant fatty acid. Studies on *T. vulgaris*, *T. serpyllum*, *T. kotschyanus*, and *T. daenensis* likewise indicate that ALA typically ranges between 40 and 65% of total seed oil, supporting the characteristic enrichment of *Thymus* seed oils in ω-3 fatty acids, particularly α-linolenic acid [16,17]. The presence of 2-hydroxy-linolenic acid as the second most abundant PUFA, although less frequently quantified in earlier works, aligns with data reported in Iranian and Mediterranean *Thymus* species, where oxygenated derivatives of linolenic acid have been suggested to play roles in seed physiology and stress adaptation [18]. Moreover, the occurrence of 2-hydroxy-linolenic acid in our sample is supported by previous findings in *T. vulgaris* seed oil, where α-hydroxylinolenic acid was identified as a naturally occurring hydroxy fatty acid representing approximately 13% of the total fatty acid fraction [19]. The relatively modest share of MUFAs (≈13%) and the predominance of oleic acid within this group are also in agreement with published data, which typically place MUFA levels between 10 and 20% in *Thymus* seeds [16,20]. Similarly, the low proportion of SFAs is characteristic of the genus, with palmitic and stearic acids representing the primary saturated fraction across most studied taxa [16,21]. Overall, the fatty acid composition observed here fits well within the chemotaxonomic ranges previously established for *Thymus*, while the notably high proportion of α-linolenic acid further supports the classification of the genus as a valuable source of ω-3 fatty acids. The high ALA content may additionally enhance the nutritional and functional value of *T. vulgaris* seed oil, considering the recognized cardioprotective and anti-inflammatory properties associated with dietary ω-3 fatty acids [21].

### 2.2. Analysis of Carotenoids

Carotenoids are recognized not only as natural pigments but also as biologically active compounds with significant antioxidant, anti-inflammatory, and health-promoting properties. Their antioxidant activity is primarily associated with the ability to quench singlet oxygen and scavenge reactive oxygen species, thereby protecting lipids, proteins, and cellular membranes from oxidative damage [22,23]. In addition to their antioxidant potential, carotenoids have been associated with modulation of immune responses, maintenance of visual function, and reduced risk of chronic diseases, particularly cardiovascular and neurodegenerative disorders [24]. Recent evidence suggests that dietary carotenoids, especially lutein and zeaxanthin, may contribute to cellular protection through the regulation of inflammatory and oxidative stress pathways [25].

Carotenoids naturally occur in numerous edible plant oils, including maize, sunflower, olive, soybean, rapeseed, or linseed oils, where they contribute not only to nutritional value but also to oxidative stability and coloration of the oils [23,26].

**Figure 1:**
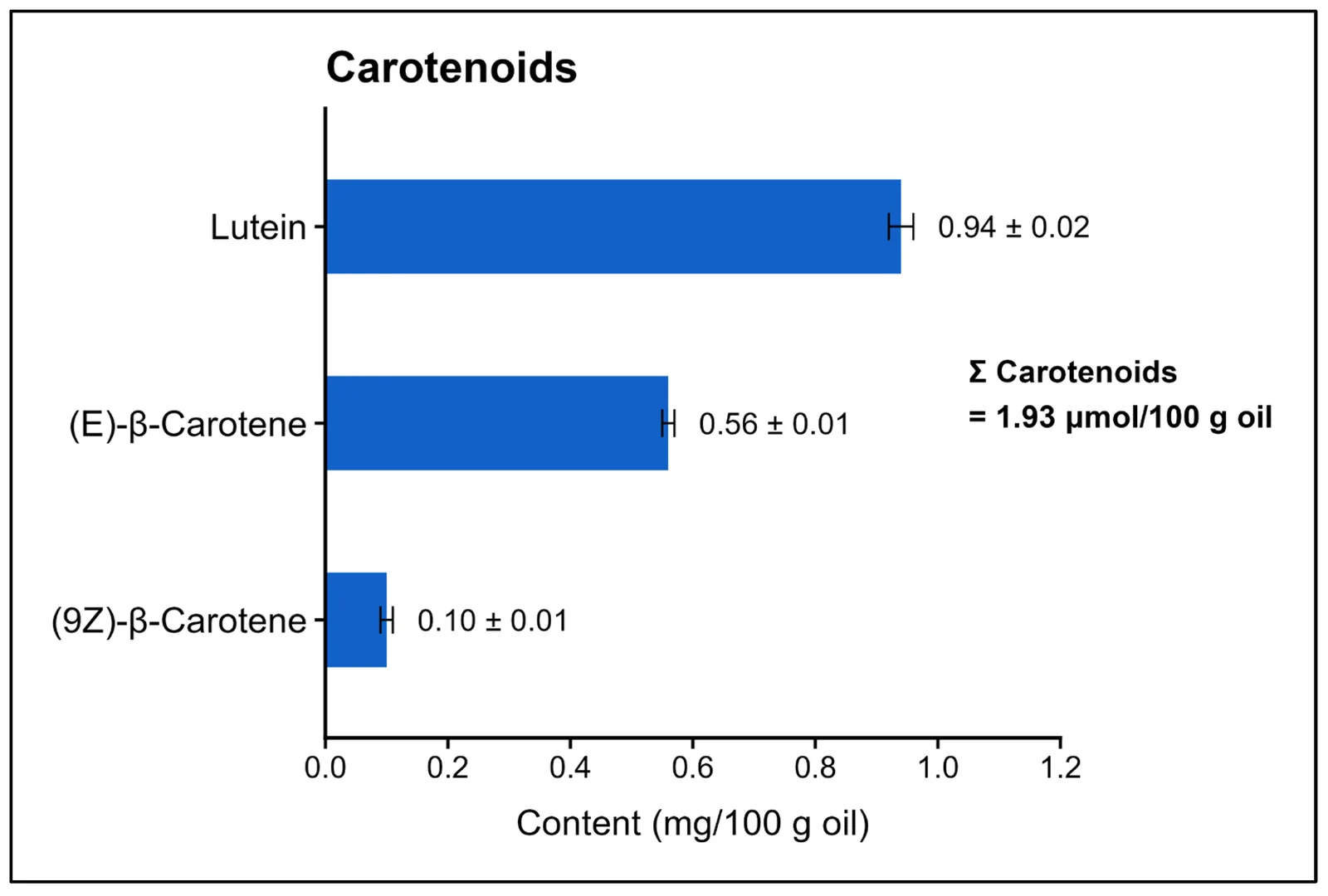
Carotenoid profile and total carotenoid content of *T. vulgaris* seed oil.

In our study, lutein was identified as the predominant carotenoid in *T. vulgaris* seed oil, followed by (E)-*β*-carotene (Figure 1). Lutein is one of the most abundant xanthophyll carotenoids in nature and is widely distributed in green leafy vegetables, flowers, fruits, and aromatic plants [27]. β-Carotene, detected as the second major carotenoid in the analyzed oil, additionally represents an important provitamin A compound and contributes to the overall antioxidant potential of plant-derived oils.

The predominance of lutein observed in our sample is in agreement with previous findings reported for species of the genus Thymus. Manukyan [28] identified lutein, zeaxanthin, chlorophyll a, chlorophyll b, α-carotene, and β-carotene as the principal carotenoid pigments in *T. transcaucasicus* Ronn., suggesting that oxygenated carotenoids are characteristic constituents of *Thymus* species. Similar carotenoid patterns rich in lutein and *β*-carotene have also been reported in other members of the family Lamiaceae, supporting the chemotaxonomic relevance of these pigments in aromatic Mediterranean plants [29].

### 2.3. Analysis of Tocols

Vitamin E comprises a group of lipid-soluble compounds commonly referred to as tocols, naturally occurring as eight structural analogues: α-, β-, γ-, and δ-tocopherols together with the corresponding tocotrienols. These compounds differ in the number and position of methyl substituents on the chromanol ring, as well as in the saturation of the phytyl side chain, with tocotrienols containing three double bonds in contrast to the saturated side chain of tocopherols [30]. Tocols are among the most important natural antioxidants in vegetable oils, where they protect polyunsaturated fatty acids from lipid peroxidation and contribute significantly to oxidative stability, nutritional quality, and the shelf life of the oils [31]. Beyond their technological role, vitamin E compounds are associated with numerous biological activities, including antioxidant, anti-inflammatory, cardioprotective, and neuroprotective effects [32].

**Figure 2:**
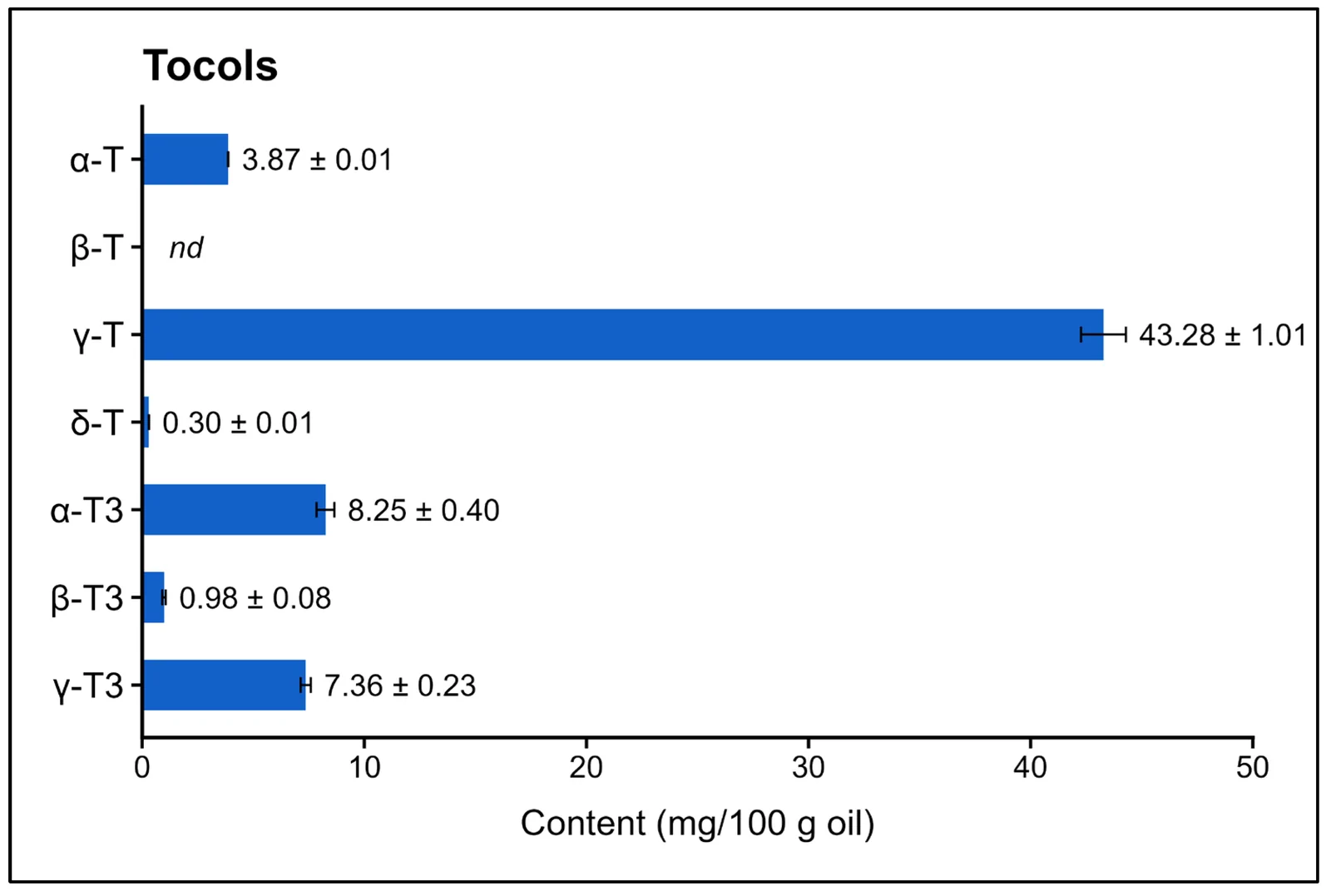
Tocol composition of *T. vulgaris* seed oil.

In our study, γ-tocopherol was identified as the predominant tocol in *T. vulgaris* seed oil, followed by γ-tocotrienol (Figure 2). The predominance of γ-homologues is characteristic of many seed oils and has previously been reported in species belonging to the family Lamiaceae. Quílez et al. [20] investigated the seed oil composition of twelve commercial Lamiaceae species and reported γ-tocopherol and γ-tocotrienol as the dominant vitamin E constituents in their samples. The total tocol content determined in our study (64.04 mg/100 g oil) is comparable to the value reported for common *T. vulgaris* seed oil by Quílez et al. [20], (70.5 mg/100 g oil).

In the same study, the authors showed that species belonging to the genus *Thymus* contained the highest amounts of γ-tocotrienol, reaching up to 18.8 mg/100 g of oil, which is 2-fold higher compared to the values obtained in our study. Such quantitative differences may be attributed to genotype, geographical origin, climatic conditions, seed maturity, or extraction methodology, all of which are known to influence tocol biosynthesis and accumulation in plant oils. Although less than in the study of Quílez et al. [20], our sample is also a good source of tocotrienols (more than 16 mg/100 g oil in total). Tocotrienols are often considered biologically more potent than the corresponding tocopherols due to their unsaturated side chain, which facilitates more efficient penetration and distribution within lipid bilayers [33]. The presence of high amounts of γ-tocopherol and γ-tocotrienol may contribute to the antioxidant capacity and biological activity of *T. vulgaris* seed oil.

### 2.4. In Vivo Hepatoprotective Activity

The liver is a central metabolic organ involved in the detoxification and biotransformation of endogenous and exogenous compounds and in the maintenance of systemic redox homeostasis. Owing to its major role in xenobiotic metabolism, the liver is particularly susceptible to oxidative damage, and oxidative stress is recognized as an important mechanism contributing to the development and progression of liver injury [34,35]. At therapeutic doses, paracetamol is predominantly metabolized through glucuronidation and sulfation, whereas a smaller fraction undergoes cytochrome P450-mediated oxidation to the reactive metabolite N-acetyl-p-benzoquinone imine (NAPQI). Under conditions of excessive paracetamol exposure, increased NAPQI formation and depletion of hepatic glutathione facilitate covalent binding of NAPQI to cellular proteins, mitochondrial dysfunction, and oxidative injury [36]. Assessment of hepatic antioxidant defense and lipid peroxidation can therefore provide insight into both the extent of paracetamol-induced oxidative disturbance and the potential protective effects of bioactive compounds.

In the present study, the effects of *T. vulgaris* seed oil (TO) on hepatic redox status were evaluated through the activities of superoxide dismutase (SOD), catalase (CAT), glutathione reductase (GR), glutathione peroxidase (GSHPx), and xanthine oxidase (XOD), together with reduced glutathione (GSH) content and lipid peroxidation (LPx) (Table 2). SOD and CAT represent important components of enzymatic antioxidant defense by limiting the accumulation of superoxide radicals and hydrogen peroxide, whereas GSH, GR, and GSHPx participate in glutathione-dependent redox regulation and peroxide detoxification. XOD can contribute to reactive oxygen species (ROS) generation under pathological conditions, while LPx reflects the oxidative degradation of membrane lipids and is widely used as an indicator of oxidative tissue injury [37]. Together, these parameters provide complementary information on hepatic redox balance and oxidative damage.

The factorial analysis revealed significant effects of TO dose and paracetamol administration, and, importantly, a significant TO dose × paracetamol interaction for all seven investigated parameters (Supplementary Table S1). The interaction effects were substantial, with partial η^2^ ranging from 0.604 for GSH to 0.988 for GR. These interactions demonstrate that the effects of increasing TO dose on hepatic redox parameters depended strongly on the presence or absence of paracetamol, rather than representing a uniform effect of the oil across experimental conditions. For parameters showing departures from model assumptions, the significance of the TO dose × paracetamol interaction was retained under HC3 heteroscedasticity-consistent inference (Supplementary Table S2), supporting the robustness of the observed factorial effects.

Paracetamol administration produced a pronounced disturbance of hepatic redox homeostasis (Table 2). Compared with untreated control animals, the paracetamol-only group showed markedly lower SOD, CAT, and GR activities and substantially depleted GSH, accompanied by higher XOD, GSHPx, and LPx values. This pattern is consistent with a strong oxidative challenge following paracetamol exposure. In particular, GSH depletion is compatible with its central role in the detoxification of NAPQI, while the pronounced increase in LPx indicates enhanced oxidative degradation of membrane lipids [37,38]. The elevated GSH-Px activity observed in the paracetamol-treated group may reflect a compensatory response to an increased peroxide burden rather than an improvement in antioxidant status. Increased XOD activity may additionally contribute to ROS generation and the amplification of the oxidative disturbance.

The response to TO differed markedly between animals with and without paracetamol exposure. In the absence of paracetamol, TO produced comparatively modest and parameter-specific changes. Formal trend analysis showed significant linear dose-related changes in SOD, XOD, CAT, GSHPx, GR, and LPx, whereas GSH showed no significant linear trend (Supplementary Table S4). Thus, administration of TO to non-intoxicated animals cannot be interpreted simply as a generalized enhancement of endogenous antioxidant capacity. Rather, the pattern suggests modulation of individual components of hepatic redox homeostasis, with considerably smaller changes than those observed under paracetamol-induced oxidative challenge.

In contrast, TO markedly modified the hepatic response to paracetamol. Planned comparisons against the paracetamol-only group showed that even the lowest TO dose (1 mL/kg b.w.) significantly affected CAT, GSHPx, GR, GSH, and LPx, whereas changes in SOD and XOD did not reach statistical significance at this dose (Supplementary Table S3). At 2 and 3 mL/kg b.w., all seven investigated parameters differed significantly from the paracetamol-only group. Increasing TO dose was associated with the recovery of SOD, CAT, GR, and GSH and with reductions in XOD, GSHPx, and LPx toward the values observed in non-intoxicated animals. In the groups receiving *T. vulgaris* seed oil alongside paracetamol, the decrease in GSH-Px activity was accompanied by increased GSH levels and GR activity, as well as a marked reduction in LPx. This concurrent pattern suggests an improvement in antioxidant defense and attenuation of lipid peroxidation, potentially reflecting a reduced need for compensatory peroxide detoxification. However, because peroxide levels and GSSG were not measured, the relative contributions of reduced oxidative challenge and changes in enzymatic capacity cannot be definitively distinguished.

The dose-related nature of this protective response was further supported by formal trend analysis. In paracetamol-treated animals, significant linear dose–response trends were observed for all seven investigated parameters (Supplementary Table S4). SOD, CAT, GR, and GSH progressively increased with increasing TO dose, whereas XOD, GSHPx, and LPx decreased. These dose–response relationships were considerably stronger in the presence of paracetamol than in non-intoxicated animals, further supporting the significant TO dose × paracetamol interactions observed in the factorial analysis. Nevertheless, the responses were not strictly proportional across all individual doses. For several parameters, particularly XOD, GSHPx, GSH, and LPx, the values observed at 2 and 3 mL/kg b.w. were relatively similar, suggesting that the response tended to approach a plateau at the higher doses. SOD and CAT, in contrast, showed further improvement at 3 mL/kg b.w. The results therefore support a clear dose-related protective response while also indicating parameter-specific differences in its magnitude and apparent saturation.

The effect on lipid peroxidation provides particularly strong evidence of attenuation of paracetamol-induced oxidative injury. LPx was significantly reduced by all three TO doses relative to the paracetamol-only group, with substantially greater reductions at 2 and 3 mL/kg b.w. (Supplementary Table S3). Because LPx reflects oxidative degradation of membrane lipids [37], its reduction suggests that TO limited oxidative membrane damage. Considered together with the recovery of SOD, CAT, GR, and GSH and the reduction in XOD and GSHPx, these findings provide convergent evidence that TO attenuated the hepatic oxidative disturbance induced by paracetamol. Importantly, this interpretation is supported not only by differences between individual experimental groups but also by the significant factorial interactions, planned contrasts, and formal dose–response analyses.

The observed protective response may be associated with the complex composition of *T. vulgaris* seed oil. The oil contains several classes of compounds with potential antioxidant activity, including tocopherols, tocotrienols, carotenoids, and polyunsaturated fatty acids. Tocopherols and carotenoids are well-established components of antioxidant defense and can protect membrane lipids by scavenging reactive species and interrupting lipid oxidation reactions [38]. In this context, the γ-tocopherol and γ-tocotrienol detected in the investigated oil may contribute to its ability to limit lipid peroxidation. Similarly, carotenoids such as lutein and β-carotene may contribute through the quenching of singlet oxygen and other reactive species. However, because the present experiment evaluated the whole seed oil rather than its isolated constituents, the observed biological response cannot be attributed to any individual compound, and the contributions of specific oil constituents remain to be elucidated.

The present results are broadly consistent with previous evidence that plant-derived antioxidants can attenuate oxidative liver injury. Ugwu and Suru [34] reviewed the hepatoprotective potential of medicinal plants in carbon tetrachloride-induced hepatotoxicity, while Kumar et al. [39] highlighted the capacity of dietary antioxidants to mitigate oxidative stress and lipid peroxidation in similar experimental models. Although carbon tetrachloride and paracetamol differ in their primary mechanisms of hepatotoxicity, these studies support the broader importance of antioxidant defense and inhibition of lipid peroxidation in protection against chemically induced liver injury.

The antioxidant and anti-inflammatory properties of *Thymus* species have also been extensively documented, particularly for phenolic-rich extracts and essential oils [40,41]. These preparations differ chemically from the seed oil investigated in the present study and therefore cannot provide direct evidence for the mechanism of the observed effect. Nevertheless, they demonstrate that *Thymus*-derived products contain multiple classes of biologically active constituents capable of modulating oxidative and inflammatory processes. Consequently, the present results extend the available evidence on *Thymus* bioactivity to the less extensively investigated seed oil fraction.

Particularly relevant to the carotenoid fraction of the investigated oil are the findings of Sindhu et al. [42], who demonstrated the hepatoprotective activity of lutein in rats exposed to paracetamol, carbon tetrachloride, and ethanol. In the paracetamol model, lutein attenuated hepatic lipid peroxidation, including reductions in lipid oxidation products, while beneficial effects on antioxidant defense were also demonstrated across the investigated models [42]. These observations are consistent with the pronounced reduction in LPx found in the present study and support the possibility that lutein may contribute to the protective properties of *T. vulgaris* seed oil. However, the contribution of lutein cannot be separated from those of tocopherols, tocotrienols, fatty acids, and other oil constituents on the basis of the present experimental design.

Overall, the factorial analysis demonstrates that *T. vulgaris* seed oil significantly modifies the hepatic oxidative response to paracetamol. The protective effect was dose-related and substantially more pronounced under paracetamol-induced oxidative challenge than under physiological conditions. At the higher TO doses, paracetamol-associated alterations in SOD, XOD, CAT, GSHPx, GR, GSH, and LPx were markedly attenuated, although complete restoration to control levels was not observed for all parameters. The combined evidence from the significant TO dose × paracetamol interactions, planned comparisons, and dose–response analyses therefore supports a hepatoprotective effect of *T. vulgaris* seed oil against paracetamol-induced oxidative injury. Modulation of endogenous antioxidant defenses and attenuation of lipid peroxidation appear to contribute to this response, while the specific contributions and potential interactions of individual seed oil constituents require further investigation.

Nevertheless, several limitations of the present study should be acknowledged. Histopathological examination of liver tissue and Western blot analysis of hepatic antioxidant enzymes were not performed, which limits the ability to confirm tissue-level changes and characterize the molecular mechanisms underlying the observed hepatoprotective effects. These additional analyses could provide further evidence to support the biochemical findings reported in this study. Future studies incorporating histopathological and molecular analyses are therefore warranted to further elucidate the mechanisms of action of *T. vulgaris* seed oil and strengthen the evidence for its hepatoprotective potential.

## 3. Materials and Methods

### 3.1. Plant Material

*Thymus vulgaris* L. seed material originated from the cultivar ‘Deutscher Winter’, initially obtained in 2008 from PHARMASAAT Arznei- und Gewürzpflanzensaatzucht GmbH (Artern, Germany), a specialized producer of medicinal and aromatic plant seeds. The original seed material was subsequently propagated and maintained for seed production at the Institute for Medicinal Plants Research “Dr. Josif Pančić” at its cultivation site in Pančevo, South Banat, Serbia.

The seed-production population was cultivated on an approximately 300 m^2^ field at a planting distance of 50 × 30 cm, corresponding to approximately 2000 plants. The plantation was renewed every four years using seed produced from the maintained population. Plants were cultivated under uniform agronomic and environmental conditions to minimize variation in seed characteristics associated with growing conditions. Seeds used in the present study were harvested from the entire seed-production population in August 2016 at full maturity. A voucher specimen of *T. vulgaris* seeds was deposited at the Institute under voucher number IPLB0816TVS. Seeds collected during the same harvest season were pooled and processed as a single production batch. Following harvest, the seeds were cleaned and graded using sieves and air separation and stored at approximately 4 °C until analysis. The moisture content determined prior to analysis was 3.44%. Immediately before extraction, the seeds were ground using an IKA MF 10 basic Microfine Mill Drive (IKA-Werke GmbH & Co. KG, Staufen, Germany).

### 3.2. Oil Extraction

The oil was extracted from 100 g of ground *T. vulgaris* seeds by Soxhlet extraction using n-hexane as the extraction solvent. Briefly, the ground seeds were placed in a cellulose extraction thimble (28 mm internal diameter × 100 mm length; Advantec, Tokyo, Japan), which was inserted into the Soxhlet apparatus. The apparatus was connected to a 250 mL round-bottom flask containing 200 mL of n-hexane. Extraction was carried out for 6 h under reflux at a temperature of 60 °C, until the solvent in the Soxhlet siphon tube became colourless. Following extraction, the solvent was removed under reduced pressure using an IKA RV 05 rotary evaporator (Staufen, Germany). The recovered oil was stored in the dark at −25 °C until further analysis. Oil content was calculated as a percentage of the dry weight (DW) of the seed material.

### 3.3. Fatty Acid Analysis

For gas chromatographic analysis, 1 g of oil was subjected to methylation using a methanolic sulfuric acid reagent to convert fatty acids into their corresponding methyl esters (FAMEs). The resulting FAME mixtures were extracted with n-hexane, and the extracts were directly analysed by gas chromatography.

FAME identification and relative composition were determined using a Shimadzu QP2010 Ultra gas chromatograph coupled to a mass spectrometer (Shimadzu Corporation, Kyoto, Japan). Chromatographic separation was performed on an InertCap 5 capillary column (60 m × 0.25 mm i.d., 0.25 µm film thickness). Helium was used as the carrier gas, with a column flow rate of 1.86 mL min^−1^ and a total flow rate of 14.2 mL min^−1^. The injector temperature was maintained at 250 °C. Samples (1 µL) were injected in split mode at a split ratio of 5:1. The oven temperature was initially set at 60 °C and held isothermally for 4 min, then increased to 280 °C at a rate of 4 °C min^−1^ and held at 280 °C for 10 min.

The mass spectrometer was operated in electron ionization (EI) mode at 70 eV. Mass spectra were acquired over the *m*/*z* range 40–400, with a solvent cut time of 7 min. The ion source and interface temperatures were maintained at 200 and 280 °C, respectively. Peak areas were integrated and the relative abundance of individual components was expressed as a percentage of the total chromatographic peak area (% RPA).

Compounds were identified by comparing their mass spectra with reference spectra from the Wiley 275, NIST/NBS, and ADAMS libraries, as well as with published literature data. Retention indices were calculated relative to an n-alkane (C_8_–C_40_) standard mixture analyzed under identical chromatographic conditions.

### 3.4. Carotenoid and Tocol Analysis

#### 3.4.1. Carotenoid and Tocol Extraction

For the analysis of the oil sample, 100 mg of oil was diluted with 1 mL methanol/tetrahydrofuran mixture (1/1, *v*/*v*). After centrifugation (5 min, 14,000 rpm), the samples were used for carotenoids analysis. For tocol analysis, 50 μL of the diluted sample was evaporated under a nitrogen stream and redissolved in 1 mL n-hexane/methyl tert–butyl ether (98 + 2, *v*/*m*). After centrifugation (5 min, 14,000 rpm), the samples were used for vitamin E analysis.

#### 3.4.2. Carotenoid Analysis

Chemical analysis of carotenoids in *T. vulgaris* oil was carried out using reversed-phase HPLC with a diode array detector at 450 nm (Merck Hitachi, Darmstadt, Germany) according to methods previously described [43]. The chromatographic separation was performed on a Develosil^®^ RPAQUEOUS C30 column (250 × 4.6 mm i.d., 5 µm particle size) (Phenomenex, Aschaffenburg, Germany). During analysis, the column temperature was maintained at 13 ± 1 °C, while the mobile phase consisted of methanol (solvent A) and methyl tert–butyl ether (solvent B). Gradient elution was performed according to the following scheme: 10–50% B 0–40 min; 50–60% B 40–42 min; 60% B 42–65 min; 60–10% B 65–70 min; 10% B 70–75 min. Flow rate was set to 1 mL/min, and the detection wavelength was 450 nm. Quantification of carotenoids was conducted based on comparison of peak areas and their specific absorption maxima with those obtained for external standards with defined concentrations, considering the recovery of the internal standard (β-apo-8′-carotinal). The concentrations of the stock solutions were checked periodically and were calculated from the specific extinction coefficients. All experiments were repeated three times.

#### 3.4.3. Tocol Analysis

Tocols were analysed using a normal-phase HPLC equipped with a fluorescence detector (excitation: 292 nm, emission: 330 nm) (Merk-Hitachi, Darmstadt, Germany) according to the previously published method [43]. An Eurospher 100 Diol analytical column (250 × 4 mm i.d., 7 µm particle size) (Knauer, Berlin, Germany) was used as the stationary phase and n-hexane/methyl tert–butyl ether (98 + 2, v/m) was used as the mobile phase with a flow rate of 1.5 mL/min. Column temperature was maintained at 30 ± 1 °C during isocratic analysis. Quantification of tocols was conducted based on comparison of peak areas with those obtained for external standards with defined concentrations, considering the recovery of the internal standard (α-tocopherol acetate). Stock solutions of each tocopherol and tocotrienol standard were prepared in ethanol and stored at −30 °C. The exact concentrations of the stock solutions were determined spectrophotometrically at their specific absorption maxima.

### 3.5. Animals and Treatments

Animal care and all experimental procedures were conducted in accordance with the Guide for the Care and Use of Laboratory Animal Resources edited by the Commission of Life Sciences, National Research Council. All procedures performed in studies involving animals were in accordance with the ethical standards of the institution (University of Novi Sad; EK: II-2017-11; 11-140-3). The animal experiments were conducted between 1 December 2017 and 1 February 2018, within the approved timeframe under Ethics Approval. The study was reported in accordance with the ARRIVE 2.0 guidelines (Animal Research: Reporting of In Vivo Experiments). The study was conducted using NMRI Hannover mice aged 4–6 weeks and weighing 20–25 g. Each experimental group consisted of six animals, including three males and three females (n = 6 per group). The animals’ health, behaviour and activity were monitored daily, while body weight was recorded weekly. Animals were fed standard mouse chow (LM2, Veterinarski zavod, Subotica, Serbia) and had free access to tap water at 25 °C and controlled humidity (30–50%) in the animal house under 12 h light/day cycles. *Thymus vulgaris* seed oil was stored at 4 °C until use and administered orally, without dilution, once daily for 7 consecutive days at doses of 1, 2, or 3 mL/kg body weight (b.w.). Hepatic injury was induced by intraperitoneal administration of paracetamol (300 mg/kg b.w.) on day 7, 24 h before sacrifice. The animals were randomly allocated to eight groups, with six mice per group, as follows: control group, which received distilled water orally once daily for 7 days; control + paracetamol group, which received distilled water orally once daily for 7 days and paracetamol (300 mg/kg b.w., i.p.) on day 7, 24 h before sacrifice; Group A, which received *T. vulgaris* seed oil at 1 mL/kg b.w. orally once daily for 7 days; Group B, which received *T. vulgaris* seed oil at 2 mL/kg b.w. orally once daily for 7 days; Group C, which received *T. vulgaris* seed oil at 3 mL/kg b.w. orally once daily for 7 days; and Groups A + paracetamol, B + paracetamol, and C + paracetamol, which received *T. vulgaris* seed oil at doses of 1, 2, and 3 mL/kg b.w., respectively, orally once daily for 7 days, followed by paracetamol administration (300 mg/kg b.w., i.p.) on day 7, 24 h before sacrifice. At the end of the experiment (day 8), 24 h after paracetamol administration, the animals were anesthetized with isoflurane, decapitated, and exsanguinated. Livers were excised immediately after sacrifice. Liver homogenates were prepared in the following way: 1 g of liver tissue was homogenized with Tris–HCl: saccharose solution (50 mmol/L; 0.25 mol/L; pH 7.40), 1:3, 4 °C, using a glass Potter–Elvehjem homogenizer set. The resulting homogenate was centrifuged at 3000 rpm for 10 min, and the concentration of proteins was determined by the biuret method using bovine serum albumin as a standard [44]. Samples of blood and liver were used for the determination of biochemical parameters of oxidative stress.

### 3.6. Biochemical Assays

The activity of xanthine oxidase (XOD) was determined using the Lück [45] method, catalase (CAT) according to Beers and Sizer [46], peroxidase (Px) according to Simon et al. [47], glutathione peroxidase (GSH-Px) according to Beutler et al. [48] and glutathione reductase (GR) according to Goldberg and Spooner [49]. The amount of reduced glutathione (GSH) was also determined according to Beutler [50], and the intensity of lipid peroxidation (LPx) was determined using the Buege and Aust [51] protocol. All measurements were performed in triplicate for each animal sample, representing technical replicates. The mean of the three measurements was used for statistical analysis, with each animal considered an independent biological replicate.

XOD activity was determined according to the method described by Lück [45] by monitoring uric acid formation during the oxidation of xanthine. Briefly, 20–50 µL of liver homogenate was added to 3 mL of a reaction mixture containing EDTA and xanthine in phosphate buffer (pH 7.5). The reaction was stopped after the specified incubation period, and the mixture was centrifuged at 3000 rpm for 10 min. Absorbance was measured at 293 nm. XOD activity was calculated using the molar extinction coefficient of uric acid (ε = 1.2 × 10^4^ L mol^−1^ cm^−1^) and expressed as nmol of uric acid formed per minute per mg of protein.

CAT activity was determined according to Beers and Sizer [46], using hydrogen peroxide (H_2_O_2_) as the substrate. The reaction mixture consisted of 20–50 µL of liver homogenate and 3 mL of 0.05 mol/L phosphate buffer (pH 7.0) containing H_2_O_2_. The H_2_O_2_-containing buffer was prepared by adding 0.075 mL of 30% H_2_O_2_ to phosphate buffer and adjusting the final volume to 50 mL. Following the reaction, the mixture was centrifuged at 3000 rpm for 10 min, and absorbance was measured at 240 nm. CAT activity was calculated using the molar extinction coefficient of H_2_O_2_ (ε = 4.36 × 10^4^ L mol^−1^ cm^−1^) and expressed relative to the protein content of the sample.

Px activity was determined according to Simon et al. [47], using H_2_O_2_ as the substrate and guaiacol as the chromogenic reagent. The reaction mixture contained 20–50 µL of liver homogenate, 3 mL of 0.1 mol/L phosphate buffer (pH 7.0), 50 µL of 0.02 mol/L guaiacol solution, and 40 µL of H_2_O_2_ solution prepared by diluting 0.14 mL of 30% H_2_O_2_ to 100 mL with distilled water. After the reaction was stopped, the mixture was centrifuged at 3000 rpm for 10 min. Absorbance was measured at 436 nm, and Px activity was calculated using the molar extinction coefficient of the reaction product (ε = 2.3 × 10^4^ L mol^−1^ cm^−1^) and expressed relative to the protein content.

GSH-Px activity was determined using cumene hydroperoxide as the substrate, following the method described by Beutler et al. [48]. Briefly, 20–50 µL of liver homogenate was mixed with 0.75 mL of 0.05 mol/L Tris-HCl buffer (pH 7.6) and incubated at 37 °C for 10 min. Subsequently, 0.1 mL of GSH solution (0.0021 mol/L) and 0.1 mL of methanolic cumene hydroperoxide solution (1:200) were added, and the mixture was incubated at 37 °C for an additional 5 min. The reaction was stopped by adding 1 mL of 20% trichloroacetic acid (TCA), followed by centrifugation at 3000 rpm for 10 min. For color development, 1 mL of the supernatant was mixed with 2 mL of 0.4 mol/L Tris-HCl buffer (pH 8.9) and 0.1 mL of DTNB solution (5,5′-dithiobis-(2-nitrobenzoic acid); 0.02 g in 5 mL of buffer). Absorbance was measured at 412 nm, and the results were calculated using the molar extinction coefficient of the reaction product (ε = 1.36 × 10^4^ L mol^−1^ cm^−1^) and expressed relative to the protein content.

GR activity was determined according to Goldberg and Spooner [49] by assessing the reduction of oxidized glutathione (GSSG) in the presence of NADPH. Liver homogenate (20–50 µL) was mixed with 0.2 mL of 2% GSSG solution, 0.3 mL of NADPH solution (0.001 mol/L), and 2 mL of phosphate buffer (pH 7.6). Following the reaction, the mixture was centrifuged at 3000 rpm for 10 min, and absorbance was measured at 340 nm. GR activity was calculated from the change in absorbance associated with NADPH oxidation and expressed relative to the protein content of the sample.

GSH content was determined according to Beutler [50] by measuring non-protein sulfhydryl groups using Ellman’s reagent. For liver samples, 1 mL of homogenate was mixed with 2 mL of 4% sulfosalicylic acid and centrifuged at 3000 rpm for 10 min. The resulting supernatant was mixed with Ellman’s reagent at a ratio of 1:40, and absorbance was measured at 412 nm. GSH content was calculated using the molar extinction coefficient of the colored reaction product and expressed relative to the protein content of the homogenate.

Lipid peroxidation was assessed by measuring thiobarbituric acid-reactive substances (TBARS) according to Buege and Aust [51]. Briefly, 20–50 µL of liver homogenate was mixed with 3 mL of reagent containing 3.75 g thiobarbituric acid (TBA), 15 g trichloroacetic acid (TCA), 20.72 mL of 37% hydrochloric acid, and one to two drops of α-tocopherol per liter of solution. The mixture was heated in a boiling water bath for 15 min and subsequently centrifuged at 3000 rpm for 10 min. Absorbance of the supernatant was measured at 535 nm. LPx levels were calculated using the molar extinction coefficient (ε = 1.56 × 10^5^ L mol^−1^ cm^−1^) and expressed as nmol/mg protein.

### 3.7. Statistical Analysis

Data are presented as mean ± standard deviation (SD). The effects of *Thymus vulgaris* seed oil (TO) dose and paracetamol administration on biochemical parameters were evaluated using two-way analysis of variance (ANOVA) in a 4 × 2 factorial design, with TO dose (0, 1, 2, and 3 mL/kg b.w.) and paracetamol administration (absent or present) included as fixed factors. The model included the main effects of TO dose and paracetamol and the TO dose × paracetamol interaction. When a significant interaction was detected, simple effects of TO dose were evaluated separately within the groups without and with paracetamol using estimated marginal means, followed by Tukey-adjusted pairwise comparisons. Effect sizes for the main effects and interaction were expressed as partial eta squared (ηp^2^). F-statistics, exact *p*-values, and effect sizes from the factorial analyses are reported in Supplementary Table S1. Statistical significance was set at *p* < 0.05.

Model assumptions were assessed by examination of model residuals, using the Shapiro–Wilk test to evaluate residual normality and Levene’s test to assess homogeneity of variances. The results of model assumption diagnostics are provided in Supplementary Table S2. LPx values were log-transformed prior to inferential analysis to improve compliance with model assumptions, whereas untransformed values are presented for descriptive purposes. Several variables showed departures from normality and/or homogeneity of variance. Therefore, HC3 heteroscedasticity-consistent inference was additionally used as a sensitivity analysis for the affected parameters. The significant TO dose × paracetamol interactions were retained under robust inference (Supplementary Table S2).

To further quantify the effect of TO on the paracetamol-induced response, planned contrasts comparing each TO + paracetamol treatment with the paracetamol-only group were estimated together with 95% confidence intervals. The corresponding *p*-values were adjusted for the three planned comparisons within each biochemical parameter using the Holm method. The results of these planned contrasts are reported in Supplementary Table S3. Linear dose–response trends were additionally evaluated separately in the absence and presence of paracetamol by treating TO dose (0, 1, 2, and 3 mL/kg b.w.) as a continuous predictor. The results of the dose–response analyses are reported in Supplementary Table S4.

All statistical analyses and graphical presentations were performed in R (R Core Team, Vienna, Austria). The ‘car’ package was used for Levene’s test and robust HC3-based inference; ‘emmeans’ for estimated marginal means, Tukey-adjusted pairwise comparisons, and planned contrasts; ‘effectsize’ for calculation of partial eta squared (ηp^2^); and ‘ggplot2’ for graphical presentation. Model fitting, factorial ANOVA, linear trend analysis, and Shapiro–Wilk tests were performed using functions available in the base R ‘stats’ package.

## 4. Conclusions

The present study provides a comprehensive characterization of *Thymus vulgaris* seed oil. The oil was characterized by a valuable lipid profile dominated by polyunsaturated fatty acids, particularly α-linolenic acid, together with appreciable amounts of bioactive carotenoids and vitamin E compounds, including lutein, β-carotene, γ-tocopherol, and γ-tocotrienol. This composition highlights *T. vulgaris* seed oil as a potentially valuable source of naturally occurring lipophilic bioactive compounds.

In vivo experiments demonstrated that *T. vulgaris* seed oil markedly attenuated paracetamol-induced oxidative disturbances in rat liver. Factorial analysis confirmed significant TO dose × paracetamol interactions for all investigated hepatic redox parameters, demonstrating that the effect of the oil depended strongly on the presence of paracetamol-induced oxidative challenge. In paracetamol-treated animals, TO administration was associated with the recovery of SOD, CAT, GR, and GSH and reductions in XOD, GSHPx, and lipid peroxidation toward the values observed in non-intoxicated animals. These effects were dose-related, with the two higher doses generally producing the most pronounced responses, although the magnitude and apparent saturation of the response differed among individual parameters.

The observed activity may be associated with the complex composition of the seed oil, including polyunsaturated fatty acids, carotenoids, tocopherols, tocotrienols, and other lipophilic constituents with potential antioxidant properties. Their individual contributions and possible interactions, however, remain to be established. Overall, the findings indicate that *T. vulgaris* seed oil attenuated selected markers of hepatic oxidative stress in an acute animal model of paracetamol-induced hepatotoxicity. Further studies are needed to elucidate the underlying mechanisms and evaluate the pharmacokinetics, bioavailability, long-term safety, and translational relevance of these findings.

## Figures and Tables

**Table 1:** Composition and classification of identified fatty acid methyl esters (FAMEs) detected in the analyzed *T. vulgaris* samples.

	Compound	Cn:m ^1^	Type ^2^	Omega Series	%RPA ^3^
1	Myristic acid	C14:0	SFA	–	0.02
2	Palmitoleic acid	C16:1	MUFA	ω-7	0.07
3	Palmitic acid	C16:0	SFA	–	4.31
4	Heptadecenoic acid	C17:1	MUFA	ω-8	2.32
5	Margaric acid (Heptadecanoic acid)	C17:0	SFA	–	0.06
6	Linoleic acid	C18:2	PUFA	ω-6	4.92
7	α-Linolenic acid	C18:3	PUFA	ω-3	63.34
8	Oleic acid	C18:1	MUFA	ω-9	10.56
9	Stearic acid	C18:0	SFA	–	2.12
10	2-Hydroxy-linolenic acid	C18:3	PUFA	ω-3	10.66
11	Eicosadienoic acid	C20:2	PUFA	ω-6	0.11
12	Gondoic acid (cis-11-Eicosenoic acid)	C20:1	MUFA	ω-9	0.47
13	Arachidic acid	C20:0	SFA	–	0.17
14	Heneicosanoic acid	C21:0	SFA	–	0.04
15	Behenic acid	C22:0	SFA	–	0.03
	Other				0.8

^1^ Cn:m—shorthand notation indicating the number of carbon atoms (n) and double bonds (m) in the fatty acid molecule. ^2^ Classification of fatty acids based on saturation level: SFA (saturated fatty acid), MUFA (monounsaturated fatty acid), and PUFA (polyunsaturated fatty acid). ^3^ Relative Peak Area, relative content of individual components calculated from their GC peak areas and expressed as percentage of the total chromatogram area.

**Table 2:** Effects of Thymus vulgaris seed oil on antioxidant defense parameters in NMRI Hannover mice with and without paracetamol treatment.

Treatment ^1^	SOD ^2,3,4^	XOD	CAT	GSHPx	GR	GSH	LPx
	(U/mg)	(nmol/mg)	(nmol/mg)	(nmol/mg)	(nmol/mg)	(nmol/mg)	(nmol/mg)
Without paracetamol							
Control	21.25 ± 0.62 ^a^	1.95 ± 0.07 ^a^	9.54 ± 0.09 ^a^	8.48 ± 0.07 ^a^	6.11 ± 0.03 ^a^	4.95 ± 0.08 ^a^	2.96 ± 0.04 ^a^
TO 1 mL/kg b.w.	20.95 ± 0.46 ^a^	1.92 ± 0.01 ^ab^	9.81 ± 0.10 ^b^	8.49 ± 0.07 ^a^	6.22 ± 0.06 ^ab^	4.75 ± 0.35 ^a^	2.94 ± 0.07 ^a^
TO 2 mL/kg b.w.	22.53 ± 0.66 ^b^	1.87 ± 0.05 ^ab^	10.13 ± 0.38 ^b^	8.13 ± 0.17 ^b^	6.36 ± 0.10 ^b^	5.07 ± 0.09 ^a^	2.48 ± 0.32 ^b^
TO 3 mL/kg b.w.	22.35 ± 1.11 ^b^	1.80 ± 0.07 ^b^	10.13 ± 0.28 ^b^	7.96 ± 0.33 ^b^	6.30 ± 0.07 ^ab^	4.95 ± 0.11 ^a^	2.68 ± 0.09 ^b^
With paracetamol							
Paracetamol	5.69 ± 0.16 ^a^	2.81 ± 0.09 ^a^	3.40 ± 0.28 ^a^	21.37 ± 0.23 ^a^	1.24 ± 0.02 ^a^	0.73 ± 0.03 ^a^	9.84 ± 0.08 ^a^
TO 1 mL/kg b.w. + P	6.39 ± 0.18 ^a^	2.83 ± 0.08 ^a^	4.01 ± 0.12 ^b^	17.82 ± 0.45 ^b^	2.13 ± 0.09 ^b^	1.35 ± 0.08 ^b^	7.98 ± 0.38 ^b^
TO 2 mL/kg b.w. + P	11.77 ± 0.74 ^b^	2.05 ± 0.04 ^b^	5.88 ± 0.27 ^c^	11.73 ± 1.11 ^c^	5.00 ± 0.20 ^c^	1.70 ± 0.19 ^c^	3.90 ± 0.33 ^c^
TO 3 mL/kg b.w. + P	13.47 ± 0.61 ^c^	2.07 ± 0.07 ^b^	6.57 ± 0.52 ^d^	10.78 ± 1.22 ^c^	5.18 ± 0.12 ^d^	1.80 ± 0.26 ^c^	3.82 ± 0.34 ^c^

^1^ TO, *Thymus vulgaris* seed oil; P, paracetamol. ^2^ SOD, superoxide dismutase; XOD, xanthine oxidase; CAT, catalase; GSHPx, glutathione peroxidase; GR, glutathione reductase; GSH, reduced glutathione; LPx, lipid peroxidation. ^3^ U, amount of enzyme required to inhibit 50% of WST reduction. ^4^ Values are presented as mean ± SD (n = 6). Different lowercase letters within the same column indicate significant differences among TO doses within each paracetamol condition (without or with paracetamol), based on simple-effects comparisons following two-way ANOVA (Tukey-adjusted *p* < 0.05). Letters should be interpreted separately for groups without and with paracetamol. LPx was analyzed after log transformation; untransformed values are presented. Complete factorial ANOVA results are provided in Supplementary Table S1.

## Data Availability

Data available on request from the corresponding author.

## Supplementary Materials

The following supporting information can be downloaded at https://www.mdpi.com/article/10.3390/plants15213404/s1. Table S1: Two-way ANOVA results for the effects of Thymus vulgaris seed oil dose and paracetamol administration on antioxidant defense parameters in Wistar rats; Table S2: Diagnostic tests for the two-way ANOVA models of antioxidant defense parameters and sensitivity analysis for GSHPx; Table S3: Planned contrasts comparing Thymus vulgaris seed oil plus paracetamol treatments with the paracetamol-only group for antioxidant defense parameters in Wistar rats; Table S4: Linear dose-response trends for Thymus vulgaris seed oil in Wistar rats in the absence and presence of paracetamol.

## Abbreviations

The following abbreviations are used in this manuscript:

TO	*Thymus vulgaris* oil
SOD	Superoxide dismutase
XOD	Xanthine oxidase
CAT	Catalase
GSHPx	Glutathione peroxidase
GR	Glutathione reductase
GSH	Reduced glutathione
LPx	Lipid peroxidation
