# Supplementary material for: Antioxidant Effects of Thymus vulgaris Seed Oil on Paracetamol-Induced Oxidative Alterations in Mice Liver Tissue
Source: Plants (Basel). 2026 Nov 2;15(21):3404. doi: 10.3390/plants15213404 (PMC13765637; doi:10.3390/plants15213404)
Supplement: Supplementary file 1 [file plants-15-03404-s001.zip › plants-4612404-supplementary/Supplementary Table S1.pdf]

Table S1. Two-way ANOVA results for the effects of Thymus vulgaris seed oil dose and paracetamol administration on antioxidant defense parameters in Wistar rats.

Parameter	Effect	df	F	p-value[1]	Partial ηp^2
**SOD**	TO dose	3, 40	157.78	3.37×10^{-22}	0.922
	Paracetamol	1, 40	4582.92	6.96×10^{-43}	0.991
	TO dose × Paracetamol	3, 40	73.28	2.65×10^{-16}	0.846
**XOD**	TO dose	3, 40	183.3	2.10×10^{-23}	0.932
	Paracetamol	1, 40	914.85	3.54×10^{-29}	0.958
	TO dose × Paracetamol	3, 40	107.53	3.47×10^{-19}	0.890
**CAT**	TO dose	3, 40	111.95	1.69×10^{-19}	0.894
	Paracetamol	1, 40	3460.88	1.81×10^{-40}	0.989
	TO dose × Paracetamol	3, 40	53.79	4.24×10^{-14}	0.801
**GSHPx**	TO dose	3, 40	215.75	9.94×10^{-25}	0.942
	Paracetamol	1, 40	1581.81	8.80×10^{-34}	0.975
	TO dose × Paracetamol	3, 40	177.61	3.77×10^{-23}	0.930
**GR**	TO dose	3, 40	1295.22	7.44×10^{-40}	0.990
	Paracetamol	1, 40	9594.06	2.91×10^{-49}	0.996
	TO dose × Paracetamol	3, 40	1056.85	4.15×10^{-38}	0.988
**GSH**	TO dose	3, 40	26.43	1.37×10^{-9}	0.665
	Paracetamol	1, 40	4624.38	5.82×10^{-43}	0.991
	TO dose × Paracetamol	3, 40	20.37	3.57×10^{-8}	0.604
**LPx**[2]	TO dose	3, 40	212.79	1.29×10^{-24}	0.941
	Paracetamol	1, 40	1488.91	2.87×10^{-33}	0.974
	TO dose × Paracetamol	3, 40	111.25	1.89×10^{-19}	0.893

[1] Values represent the results of two-way ANOVA with TO dose (0, 1, 2, and 3 mL/kg b.w.) and paracetamol administration (absent or present) as fixed factors. Numerator and residual degrees of freedom are reported as df. Effect sizes are expressed as partial eta squared (ηp^2).
[2] LPx was analyzed after log transformation.
