# Supplementary material for: Antioxidant Effects of Thymus vulgaris Seed Oil on Paracetamol-Induced Oxidative Alterations in Mice Liver Tissue
Source: Plants (Basel). 2026 Nov 2;15(21):3404. doi: 10.3390/plants15213404 (PMC13765637; doi:10.3390/plants15213404)
Supplement: Supplementary file 1 [file plants-15-03404-s001.zip › plants-4612404-supplementary/Supplementary Table S2.pdf]

Table S2. Diagnostic tests for the two-way ANOVA models of antioxidant defense parameters and sensitivity analysis for GSHPx.

A. Model assumption diagnostics

Parameter	Shapiro–Wilk W[2]	p-value	Levene's F	p-value
SOD	0.959	0.0922	1.979	0.0823
XOD	0.983	0.6971	1.437	0.2181
CAT	0.961	0.1113	4.283	0.0013
GSHPx	0.904	0.0009	8.805	1.63×10^{-6}
GR	0.962	0.1268	1.968	0.0839
GSH	0.939	0.0149	2.793	0.0183
LPx[1]	0.929	0.0062	3.656	0.0038

B. GSHPx sensitivity analysis using HC3 robust covariance estimation

Parameter	Robust F	df	p-value
CAT	41.94	3, 40	2.01×10^{-12}
GSHPx	256.69	3, 40	3.73×10^{-26}
GSH	46.38	3, 40	4.33×10^{-13}
LPx[1]	181.73	3, 40	2.47×10^{-23}

[1] LPx diagnostic tests were performed on log-transformed data used for inferential analysis.
[2] Shapiro–Wilk tests were applied to model residuals, and Levene's tests were used to assess homogeneity of variances across the eight treatment combinations. Parameters showing a significant departure from residual normality and/or homogeneity of variance ($p < 0.05$) were additionally subjected to sensitivity analysis using an HC3 heteroscedasticity-consistent covariance estimator. Robust F-statistics are reported for the TO dose × paracetamol interaction, which was the primary effect of interest.
