# Supplementary material for: Antioxidant Effects of Thymus vulgaris Seed Oil on Paracetamol-Induced Oxidative Alterations in Mice Liver Tissue
Source: Plants (Basel). 2026 Nov 2;15(21):3404. doi: 10.3390/plants15213404 (PMC13765637; doi:10.3390/plants15213404)
Supplement: Supplementary file 1 [file plants-15-03404-s001.zip › plants-4612404-supplementary/Supplementary Table S3.pdf]

Table S3. Planned contrasts comparing *Thymus vulgaris* seed oil plus paracetamol treatments with the paracetamol-only group for antioxidant defense parameters in Wistar rats.

Parameter	Contrast	Estimate[1]	95% CI [2]	Adjusted p-value 3
**SOD**	TO1 + P vs P	0.705	−0.038 to 1.448	0.0623
	TO2 + P vs P	6.082	5.339 to 6.825	3.46×10^{-19}
	TO3 + P vs P	7.782	7.039 to 8.525	7.33×10^{-23}
**XOD**	TO1 + P vs P	0.025	−0.050 to 0.100	0.5019
	TO2 + P vs P	−0.755	−0.830 to −0.680	2.52×10^{-22}
	TO3 + P vs P	−0.743	−0.818 to −0.669	2.97×10^{-22}
**CAT**	TO1 + P vs P	0.615	0.338 to 0.892	6.02×10^{-5}
	TO2 + P vs P	2.48	2.125 to 2.835	1.15×10^{-16}
	TO3 + P vs P	3.172	2.638 to 3.705	1.53×10^{-14}
**GSHPx**	TO1 + P vs P	−3.550	−4.008 to −3.092	1.12×10^{-18}
	TO2 + P vs P	−9.633	−10.653 to −8.613	2.89×10^{-21}
	TO3 + P vs P	−10.590	−11.708 to −9.472	2.89×10^{-21}
**GR**	TO1 + P vs P	0.885	0.767 to 1.003	3.60×10^{-18}
	TO2 + P vs P	3.755	3.637 to 3.873	1.07×10^{-41}
	TO3 + P vs P	3.938	3.820 to 4.056	2.43×10^{-42}
**GSH**	TO1 + P vs P	0.623	0.544 to 0.703	2.38×10^{-18}
	TO2 + P vs P	0.973	0.803 to 1.144	5.44×10^{-14}
	TO3 + P vs P	1.073	0.841 to 1.306	1.41×10^{-11}
**LPx[4]**	TO1 + P vs P	0.81	0.776 to 0.847	4.88×10^{-12}
	TO2 + P vs P	0.396	0.365 to 0.428	9.65×10^{-25}
	TO3 + P vs P	0.387	0.357 to 0.420	9.65×10^{-25}

[1] Estimates represent differences between each TO + paracetamol treatment and the paracetamol-only group (TO + P − P), with positive values indicating an increase and negative values a decrease relative to the paracetamol-only group.

[2]For parameters showing departures from model assumptions, HC3 heteroscedasticity-consistent inference was used.

[3] P-values were adjusted for the three planned comparisons within each biochemical parameter using the Holm method.

[4]LPx was analyzed on the log scale, and estimates and 95% confidence intervals are presented as back-transformed ratios (TO + P/P).
