# Supplementary material for: Antioxidant Effects of Thymus vulgaris Seed Oil on Paracetamol-Induced Oxidative Alterations in Mice Liver Tissue
Source: Plants (Basel). 2026 Nov 2;15(21):3404. doi: 10.3390/plants15213404 (PMC13765637; doi:10.3390/plants15213404)
Supplement: Supplementary file 1 [file plants-15-03404-s001.zip › plants-4612404-supplementary/Supplementary Table S4.pdf]

Table S4. Linear dose-response trends for Thymus vulgaris seed oil in Wistar rats in the absence and presence of paracetamol.

Parameter	Paracetamol	Slope per 1 mL/kg TO[1]	95% CI	p-value	R^2
SOD	Absent	0.488	0.172 to 0.805	0.0041	0.318
	Present	2.872	2.451 to 3.294	1.63×10^{-12}	0.901
XOD	Absent	−0.051	−0.071 to −0.030	3.47×10^{-5}	0.549
	Present	−0.301	−0.376 to −0.226	3.19×10^{-8}	0.758
CAT	Absent	0.209	0.115 to 0.303	1.32×10^{-4}	0.493
	Present	1.138	0.978 to 1.298	7.26×10^{-13}	0.908
GSHPx	Absent	−0.190	−0.267 to −0.113	3.88×10^{-5}	0.544
	Present	−3.785	−4.312 to −3.259	5.48×10^{-13}	0.91
GR	Absent	0.071	0.039 to 0.103	1.32×10^{-4}	0.493
	Present	1.469	1.246 to 1.691	3.05×10^{-12}	0.895
GSH	Absent	0.034	−0.048 to 0.116	0.4024	0.032
	Present	0.357	0.278 to 0.436	3.59×10^{-9}	0.801
LPx[2]	Absent	−0.048	−0.080 to −0.015	0.0059	0.297
	Present	−0.357	−0.418 to −0.295	3.60×10^{-11}	0.869

[1] Linear dose-response trends were evaluated separately in the absence and presence of paracetamol by treating TO dose (0, 1, 2, and 3 mL/kg b.w.) as a continuous predictor. Slopes represent the estimated change in the respective parameter per 1 mL/kg increase in TO dose. R^2 represents the proportion of variance explained by the linear dose-response model.

[2]LPx was analyzed after log transformation; therefore, the reported slope and 95% confidence interval for LPx are expressed on the log scale.
